# Round the Hospitals

**Published:** 1921-04-02

**Authors:** 


### ROUND THE HOSPITALS.

The report of the meeting of the General Nursing Council, which we give on page 21, is full of interest both for what it includes and for what it omits. Chief among the latter is the discussion on the opinion of the Law Officers of the Crown in regard to matters which have been submitted to them. It will be remembered that the rules when drafted by the Council have to be sanctioned by the Minister of Health, and it was hoped to have had his sanction before now, seeing that they were sent down to him six months ago. Instead of sanction comes "the opinion of the Law Officers of the Crown," which the Council decided to discuss *in camerâ*. There are, as the records of proceedings of the two other Councils indicate, several delicate matters in connection with the rules, including the question of reciprocal registration and the qualification of existing nurses. Concerning the latter, the Council for Ireland reported at its last meeting that legal opinion has been taken which has empowered the acceptance of one year's hospital training. We agree with Mrs. Bedford Fenwick in her insistence at the Council meeting on March 24 that the less the Council's proceedings are *in camerâ* the better, and that if the rules as approved are not to the best benefit of the profession then the nurses should have the whole case placed before them. It is to be regretted that facilities for this ample knowledge were not available at that meeting in connection with the discussions on the reports of the Registration and Education and Examination Committees, which were denied to the Press, though their provisions and recommendations were lengthily debated. It is manifestly impossible to give an intelligible report of discussions based on draft rules which are not available. So long as these meetings are open to the Press courtesy requires that the facilities for an adequate report should be ample.

The remains of the late Mr. Henry Bonham-Carter, Florence Nightingale's cousin and coadjutor in the Nightingale Training-School, whose death we recorded last week, were laid to rest on Saturday, March 26, at the family burying-place, Burlton, in Hampshire. On Tuesday last a memorial service was held in St. Thomas's Chapel, and was largely attended by members of Mr. Bonham-Carter's family, governors and staff of St. Thomas's Hospital, and the staff of the Nightingale Training-School, as well as by representatives of many branches of nursing. The service was conducted by the Rev. H. F. S. Adams, vicar of St. John's, Southwick Crescent, assisted by the Rev. W. S. Thomas, chaplain of St. Thomas's Hospital.

An article in the *Church Times*, seeming to attribute the scarcity of nurses in London hospitals to "the high wage offered by local authorities to uneducated and untrained women to be nurses in Poor-law infirmaries," has brought forth

**ROUND THE HOSPITALS**—(*continued*).

a spirited rejoinder from Miss Barton as President of the Poor-law Infirmary Matrons' Association. Miss Barton deprecates the slur cast upon the Poor-law service by the imputation that those who enter it are "uneducated," and pleads that the work they have to do during their training is the same as in the general hospitals. She urges that it is equally important, though perhaps more difficult, to attract the best type of women to enter for training in the Poor-law infirmaries where their presence is so much needed." Another writer, "Cornubia," in the same issue efficiently takes up the cudgels on behalf of the Poor-law nurses and the infirmaries where they minister. Blessed are the uses of advertisement, and the infirmaries are to be congratulated that occasion has been given for emphasising the truth about them. May it bring in its wake a good crop of candidates.

Astonishingly few nurses with fever training only are found entered on the Fever Register of the Scottish Board of Health. Out of 1,137 nurses, 622 have received further training in several hospitals and infirmaries. Of the remaining 515, nearly 100 are to be deducted as dead or not practising, and this figure should probably be much larger. One of the awkward contingencies attaching to fever nursing is the very large number of young women who take service for a year or two, or perhaps a few months only, to fill in time, without the smallest intention of taking training. The managers of large fever institutions are compelled in times of epidemic to engage many extra "hands," but they cannot instil into these employees the desire to embrace the career of a nurse. Out of 1,000 candidates obtained last autumn by the M.A.B. to meet the scarlet-fever epidemic, only 10 per cent. were willing to enter as probationers, even for a two-year course.

Leicestershire is leading the way in a much-needed co-ordination of isolation hospital nursing. The usual method pursued in country isolation hospitals is to engage one or two nurses and let them wrestle single-handed, or with one or two so-called probationers of the charwoman order, until the number of patients exceeds a fixed limit. Thereupon private nurses are engaged at some three and a-half guineas a week, and do not have a very happy time. The new plan adopted in Leicestershire provides for a central committee dealing with the nine isolation hospitals in the county, with a view to securing efficient nursing and suitable accommodation in all districts. A scheme of mobile nursing is to be introduced, under which nurses will take duty at any hospital as required. When an epidemic occurs in one of the districts nurses will be transferred to it from hospitals where the number of patients is low, and it is hoped that the unsatisfactory and costly expedient of engaging private nurses for isolation work will be avoided. We hear that in some counties the standard of nursing in isolation hospitals is below what would be thought credible.

The annual report of the Royal Southern Hospital, Liverpool, is memorable from the fact that it chronicles the opening, at the end of 1920, of the Nurses' Home extension, built and equipped on the most modern and up-to-date manner from the plans of Mr. I. W. Haigh, architect. It provides accommodation for twenty-one additional nurses, and enables the Committee "to reduce the working hours of the staff, increase off-duty and holiday periods, and so give to the nurses a maximum respite from their arduous duties." A room in the Nurses' Home has been renovated and adapted as a writing-room at a cost of £20, the gift of one of the Governors.

The Ministry of Pensions announces that disabled nurses intending to claim alternative retired pay or pension based on pre-war earnings and present earning capacity must prove their pre-war earnings before July 2, 1921, or within one year since the first award of disability, retired pay or pension, whichever is the later date. A disabled nurse is not eligible for alternative pension if her pre-war earnings did not exceed £95 a year.

The directors of the Royal Hospital for Sick Children, Aberdeen, are starting a pension scheme for their nurses, who will have the option of joining it when "signing-on" after their two months' probation. The scheme takes the form of a pension of £10, payable at the age of fifty-five, in the Royal National Pension Fund for Nurses, half of the premium being paid by the hospital and half by the nurses. As nurses in children's hospitals are, as a rule, very young, this means only a very small deduction from their salary. The beginning of an effort to save in early professional years is of incalculable value, far exceeding the direct benefit of so small a pension, and is worth encouragement.

The claim of nurses through the District Nursing Associations to some of the accumulated surplus of the approved societies is very strong. By far the greater number of the nurses' patients in many localities are insured persons, and hitherto the work of bringing skilled nursing into the homes of the insured has been carried out free of charge to those most nearly concerned. In innumerable cases it can be shown that the societies have benefited financially to a considerable extent by the labour of the nurses. Cases which without careful nursing might remain on the books for many weeks quickly yield to treatment carried out by the nurses under doctor's orders. It is the impecuniosity of the District Nursing Associations which forces down the pay of nurses in this branch of work. Were a reasonable capitation fee payable by the societies for their insured members who require nursing aid a great impetus would be given to this branch of work, and the nurses could be accorded remuneration on a more equitable scale.

A general conference is now in progress at Geneva, under the auspices of the International Red Cross Committee, at which delegates from many

**ROUND THE HOSPITALS**—(*continued*).

countries are assisting. This Committee claims to have inaugurated the measures for combating typhus in Middle and Eastern Europe, and it has carried out good work in various directions for the relief of the populations affected by war. The League, which embraces all the Red Cross Societies of the Entente countries and the neutral Red Cross Associations, is about to collaborate with this Committee in the task of helping the Russian refugees in Constantinople. The Committee consists exclusively of Swiss citizens resident in Geneva.

---

Speaking before the London Centre of the College of Nursing, Sir Robert Armstrong-Jones stated that he formerly held strong opinions on the desirability, and indeed the necessity, for the mental nurse to be also a trained hospital nurse. But as hospital training is of so specialised a character and has become of late much more so, embracing many branches, he now feels somewhat convinced, since the human span of life is limited, that it is unfair in these competitive days to force the mental nurse to be also a hospital nurse. It is the true spirit of nursing that we need in mental hospitals irrespective of the particular curriculum. Before the war there were some 20,000 persons employed in the service of the insane in this country, of whom some 13,000 male and female nurses held the certificate of the Medico-Psychological Association. It is very doubtful if the proportion of trained mental nurses is as great to-day.

---

A great scarcity of nurses exists at present in the State of Victoria. Especially does the Bush Nursing Association find it difficult to staff its thirty-seven centres. The reasons for this special scarcity are obvious—nurses will not go into the bush; the responsibilities are too great and the pay too small for the hardships that have to be endured. The bush nurse must be qualified in all branches; she must be able to ride and drive, and is required to ride and drive alone anywhere, at any hour, and in all weathers. In midwifery cases the nurse is saddled with all the responsibility, miles from medical aid, and in nearly all cases with no conveniences of any sort. It has been seriously proposed that women expecting confinement should be compelled by law to come to the nearest township to the nurse, and the suggestion has much to recommend it, as nowadays even small places have a doctor, and also conveniences that cannot be had in the back blocks, where the nurse has to take two lives in her hand. Another present scarcity is midwifery nurses, but as regards Victoria that is to be expected as long as that State pursues the present narrow-minded policy of requiring all nurses who come here, whether from other States or from the Mother Country, to pass the examination of the Royal Victorian Trained Nurses Association, Melbourne, before they can be registered. It recently happened that a nurse holding the certificate of the London Hospital, and also that of the London Central Midwives Board, found it difficult to secure registration in Victoria. The certificate of the Australian Trained Nurses Association (New South Wales), absolutely equivalent to that of the R.V.T.N.A., is not recognised in Victoria; the applicant has to sit for the local examination before registration can be secured.

